# Incomplete removal of *Wolbachia* with tetracycline has two-edged reproductive effects in the thelytokous wasp *Encarsia formosa* (Hymenoptera: Aphelinidae)

**DOI:** 10.1038/srep44014

**Published:** 2017-03-07

**Authors:** Xiao-Xiang Wang, Lan-Da Qi, Rui Jiang, Yu-Zhou Du, Yuan-Xi Li

**Affiliations:** 1 Department of Entomology, Nanjing Agricultural University, Nanjing, Jiangsu 210095, China; 2 Institute of Applied Entomology, School of Horticulture and Plant Protection, Yangzhou University, Yangzhou, Jiangsu 225009, China

## Abstract

*Wolbachia pipientis* are intracellular endosymbionts that induce parthenogenesis in the parasitoid *Encarsia formosa*. Previous studies that focused on effects of *Wolbachia* on the wasp usually used tetracycline to remove *Wolbachia* without concern for the joint influences of tetracycline and *Wolbachia*. Here we treated the wasps (F0 lines) with tetracycline to produce offspring (F1 lines) which were not fed tetracycline to avoid antibiotic influence. The quantitative data and fluorescence *in situ* hybridization showed that *Wolbachia* titers were reduced but not totally removed. The *Wolbachia* that infected the male offspring were unpredictably detected. Low dose tetracycline enhanced the fertility of 2-day-old F0 wasps after 24 h of treatment; however, compared with controls, the oocyte load of 3- to 6-day-old tetracycline-treated wasps decreased day by day, and tetracycline reduced the longevity of the wasps. The fecundity of controls was significantly higher than that of the treated F1-10 and F1-20 generations. Gene expression of vitellogenin reflected the same trend as that of wasp fecundities in both F0 and F1 lines. Moreover, female offspring proportions of F0 and F1 lines were related to the titer of infected *Wolbachia*, demonstrating that *Wolbachia* titer affected the sex determination of *E. formosa*.

*Wolbachia pipientis* are endosymbiotic bacterium that infects at least 40% of insect species and vertically transmitted through the host egg^1^. *Wolbachia* modify host biology in various ways, including the induction of reproductive manipulations such as cytoplasmic incompatibility (fertilized eggs from cross between bacteria-infected male and uninfected-female can not hatch) and thelytokous parthenogenesis^2^,^3^,^4^,^5^, in which the infected females rarely produce males and diploid females develop from their unfertilized eggs. *Wolbachia* not only manipulate host reproduction but also affect host fitness including fecundity. It was found that infection with a native *Wolbachia* is associated with a higher fecundity, which was indicated by the number of offspring, in *Trichogramma bourarachae* than uninfected counterparts^6^. In contrast, arrhenotokous lines of *T. deion, T. pretiosum* and *T. cordubensis*, which were obtained by feeding antibiotics to *Wolbachia*-infected lines for several generations, produced more offspring than their thelytokous counterparts^7^,^8^. Moreover, effects of *Wolbachia* on the reproductive biology of their hosts depend on combination of *Wolbachia*-host. For instance, in combination of *Wolbachia*-*T. bourarachae*, the fertility of the naturally *Wolbachia*-infected *T. bourarachae* was enhanced compared with the uninfected natural one, but neither thelytoky nor CI was induced^6^; in combination of *Wolbachia-T. deion*, not only lower fecundity but also thelytoky in host was induced by infection of *Wolbachia*^7^. Additionally, no negative effects of *Wolbachia* density on reproductive fitness of the host were found when obligate parthenogenesis was induced in *Muscidifurax uniraptor* by *Wolbachia*^9^. Therefore, the reproductive effects of *Wolbachia* are variable.

*Encarsia formosa* Gahan (Hymenoptera: Aphelinidae) is an endoparasitoid of the whitefly (*Bemisia tabaci* Gennadius)^10^ and world widely used as a biological control agent of whiteflies in green houses^11^. The thelytokous reproduction of the parasitoid is induced by *Wolbachia*^12^. A few studies have investigated the influence of *Wolbachia* on fecundity by using certain concentrations of antibiotics to treat *E. formosa*; however, the results varied^12^,^13^,^14^,^15^. Some results showed that treated *E. formosa* produced more offspring than the untreated ones did^12^,^14^. In contrast, significant reductions in the number of progeny from treated *E. formosa* have also been observed^13^,^15^. However, the short or long-term influences of tetracycline on reproduction were not considered, and only one generation of wasps was investigated in the studies^12^,^13^,^14^. Usually, comparisons of infected hosts with uninfected ones should be carried out under identical genetic backgrounds to determine the effect of an endosymbiont on host fitness^10^,^16^; however, sexual *E. formosa* lines cannot be established by treating individuals with antibiotics^13^. It is suggested that the invasion of *Wolbachia* occurred long time ago and the infection has been fixed^12^. Moreover, in these studies, it was unclear whether *Wolbachia* were completely eliminated because the *Wolbachia* density was not tested in either female or male offspring^12^,^13^. The number of offspring was used as the main index in these studies. In fact, *Wolbachia* mainly resided in reproductive tissues and affected oocyte development, which was correlated to the offspring numbers^17^,^18^. Therefore, an alternative index is needed to evaluate the effect of *Wolbachia* on the fecundity of this parasitoid.

Vitellogenin is a fundamental element in the reproductive machinery of female insects. It provides nutrition for egg development^19^,^20^. During vitellogenesis, the fat body produces mostly yolk protein precursors which are secreted into the haemolymph and accumulate in the developing oocytes during oogenesis^19^,^21^,^22^,^23^. Because vitellogenin synthesis is positively correlated with egg maturation, embryonic growth and oviposition capacity^24^,^25^,^26^, the quantity of vitellogenin is usually used as a critical role in reproductive assessment^27^,^28^,^29^,^30^,^31^. Therefore, determining the expression levels of vitellogenin is one approach for elucidating changes in insect reproduction. Furthermore, because the genome of *E. formosa* contains only one vitellogenin gene, this approach becomes more feasible^20^.

In this study, because tetracycline caused high mortality of treated individuals, and males lost function of fertilizing female together with high male ratio in offspring, we failed to establish *Wolbachia*-uninfected lines of *E. formosa*. Simultaneously, we treated *E. formosa* (F0 lines) with different concentrations of tetracycline to produce F1 lines with low *Wolbachia* titers and investigated the quantity of *Wolbachia*, the oocyte load, vitellogenin gene expression of *E. formosa* and its rate of parasitism on whitefly nymphs to clarify the effects of *Wolbachia* and tetracycline on wasp reproduction. We also found *Wolbachia* infections not only in female but also in male offspring of treated wasps by using PCR and fluorescence *in situ* hybridization (FISH).

## Results

### Attempt at establishing a *Wolbachia*-uninfected line of *E. formosa*

**Figure 1:**
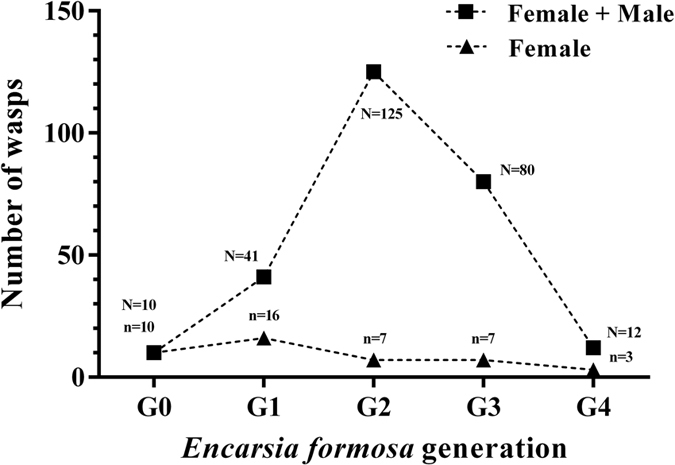
The number of offspring produced by *E. formosa* wasp in each generation. G0 denotes the parental generation, while G1- G4 denote the filial generations. G0, G1, G2, and G3 were all treated with 10 mg/ml tetracycline solution. N: number of the total wasps; n: number of the female wasps.

Ten treated female wasps from the parent generation (G0) produced 41 offspring that included 16 females (G1) (Fig. 1). After being treated with tetracycline, these 16 females produced seven females and 118 males (G2). The G2 generation was the largest number of offspring wasps in five generations, but the number of wasps did not increase over the next two generations. The G3 generation produced seven females and 73 males, but the G4 generation produced only three females and 12 males. Unfortunately, the female wasps were still infected by *Wolbachia* in these generations, even though every generation was treated with tetracycline (Figure S1). The three females in G4 did not produce female offspring and the effort to establish a *Wolbachia*-uninfected line eventually failed.

### Quantification of *Wolbachia*

**Figure 2:**
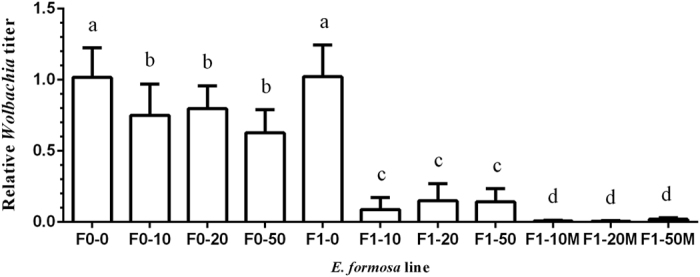
Relative *Wolbachia* titers in *E. formosa*. F0-0, F0-10, F0-20, and F0-50 were lines treated with 0, 10, 20, and 50 mg/ml tetracycline, respectively. *Wolbachia* titers were measured 1 d later. The F1-0, F1-10, F1-20, and F1-50 lines were female offspring of F0-0, F0-10, F0-20, and F0-50 wasps, respectively. M denotes males. The values are means + SD, means with different letters are significantly different (LSD test, *P* < 0.05).

*Wolbachia* titers in F1 lines, whether males or females, were significantly lower than that in F0 lines, except for that in F1-0 line (Fig. 2, F_10,88_ = 78.04, *P* < 0.001). *Wolbachia* titers in F0-10, F0-20, and F0-50 females were not significantly different, but all were lower than that in line F0-0 (Fig. 2, F_3,32_ = 6.65, *P* < 0.0001). Within the F1 lines, *Wolbachia* titers in F1-10, F1-20, and F1-50 were all significantly lower than those in the F1-0 line (Fig. 2, F_6,56_ = 103.89, *P *<* *0.0001), and *Wolbachia* titers in females were higher than those in males (Fig. 2, F_5,48_ = 7.94, *P* < 0.0001).

### FISH detection of *Wolbachia*

**Figure 3:**
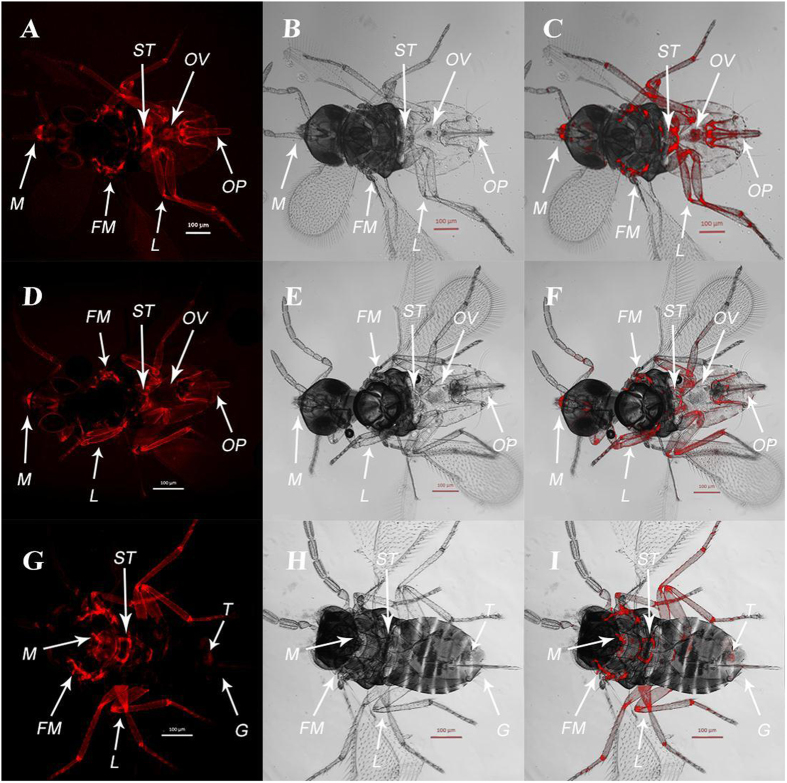
FISH detection of *Wolbachia* in females and males of *E. formosa.* (**A,B,C**) An F0-0 unfed female. (**D,E,F**) An F1-10 female offspring of F0-10 that was treated with 10 mg/ml tetracycline. (**G,H,I**) An F1-10 male offspring of F0-10 that was treated with 10 mg/ml tetracycline. *M*: mouthpart. *FM*: fight muscles. *L*: legs. *ST*: somatic tissues. *OV*: ovary. *OP*: ovipositor. *T*: testis. *G*: genitalia. Left panels (A,D,G) fluorescence signals; middle panels (B,E,H) bright field; right panels (C,F,I) combined bright field and fluorescence.

*Wolbachia* was found in the mouthparts, bilateral flight muscles, somatic tissues, six legs and reproductive systems of all tested control (F0-0) *E. formosa* females (Fig. 3A–C). Although the F1-10 females were infected with *Wolbachia* in the mouthparts, bilateral flight muscles, somatic tissues and six legs, no fluorescence signals of *Wolbachia* were found in the ovaries (Fig. 3D–F). In two out of three F1-10 males tested, *Wolbachia* were detected in the heads, chest muscles, legs, and the undersides of the bodies, where the reproductive tissues are located, one with strong signal was presented in Fig. 3G,I.

### Parasitism rate, oocyte load and vitellogenin gene expression of F0 generation

**Figure 4:**
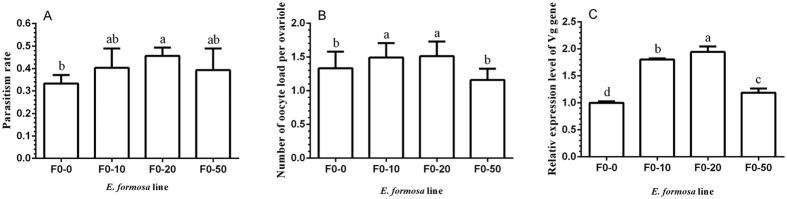
The reproductive effects of tetracycline on F0 wasps. (**A**) Parasitism rate. (**B**) Oocyte load per ovariole. (**C**) Relative expression levels of the vitellogenin gene. F0-0, F0-10, F0-20, and F0-50 denote lines treated with 0, 10, 20, and 50 mg/ml tetracycline, respectively. The values are means + SD; means with different letters are significantly different (LSD test, *P *<* *0.05).

**Figure 5:**
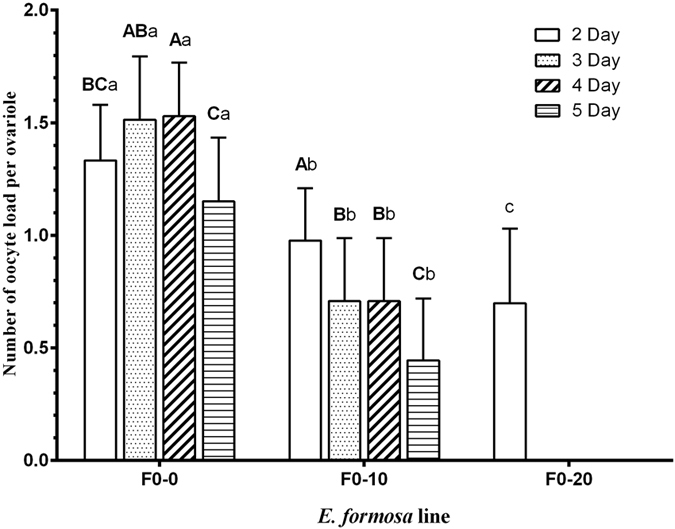
The effects of tetracycline on the oocyte load of wasps. The F0-0, F0-10, and F0-20 lines were treated continuously with 0, 10, and 20 mg/ml tetracycline, respectively. The values are means + SD; means with different letters are significantly different (LSD test for 2 day among F0-0, F0-10 and F0-20; *t* test for other ages between F0-0 and F0-10; *P *<* *0.05). The uppercase letters indicate comparisons among different ages of the same line; the lowercase letters indicate the comparisons among different lines of the same age.

The parasitism rates of whitefly nymphs by F0-0, F0-10, F0-20, and F0-50 wasps were 33.33 ± 3.80%, 40.27 ± 8.67%, 45.60 ± 3.70%, and 39.33 ± 9.57%, respectively. Compared with control (F0-0), slight increases in both treatments of 10 mg/ml and 50 mg/ml and a significant increase in treatment of 20 mg/ml in parasitism rate occurred (Fig. 4A, F_3,16_ = 1.73, *P *<* *0.05). Oocyte load per ovariole of the two-day-old F0-10 and F0-20 wasps were 1.5 ± 0.2 and 1.5 ± 0.2, respectively, both of which were significantly higher than the oocyte load per ovariole of the F0-50 (1.2 ± 0.2) and control (1.3 ± 0.2) lines (Fig. 4B, F_3,56_ = 10.29, *P *<* *0.0001). Compared with the control line, the relative expression levels of the vitellogenin gene of treated lines were significantly higher, and the vitellogenin gene expression level of 2-day-old F0-20 wasps was the highest among all lines (Fig. 4C, F_3,8_ = 146.64, *P* < 0.0001). Continuous feeding of adults with tetracycline solution caused a significant decrease in oocyte load of the females than that of control (F0-0) in subsequent days (Fig. 5, 3-day-old: F_2.42_ = 20.314, *P* < 0.0001; 4-day-old: *t* = 7.88, *P* < 0.0001; 5-day-old: *t* = 8.65, *P* < 0.0001; 6-day-old: *t* = 6.95, *P* < 0.0001), and oocyte load of F0-10 females decreased significantly day by day (F_3,56_ = 9.94, *P* < 0.001).

### Longevity of F0 generations

**Figure 6:**
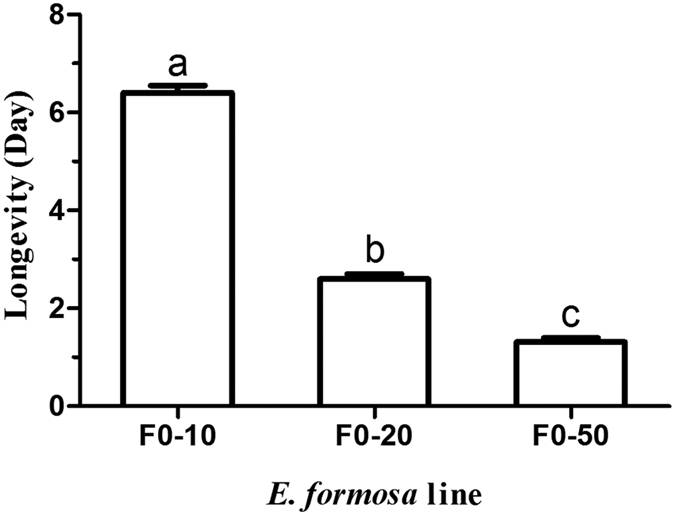
Longevity of F0 generation with continuous feeding on tetracycline. The values are means + SD; means with different letters are significantly different (LSD test, *P *<* *0.05).

To further investigate the toxicity of tetracycline on *E. formosa*, the F0 lines were continuously treated with tetracycline solution until the wasps died. The longevity decreased significantly with the increase of tetracycline concentration (Fig. 6, F_2,132_ = 322.54, *P *<* *0.0001). The F0-10 wasps survived for 6.42 ± 1.01 days. The F0-20 wasps lived 2.60 ± 0.69 days, while the F0-50 wasps lived only 1.31 ± 0.60 days. All females in treatments with tetracycline died before the eighth day, on the contrary, all 45 females in control (F0-0) were still alive.

### Parasitism rate, oocyte load and vitellogenin gene expression of the F1 generation

**Figure 7:**
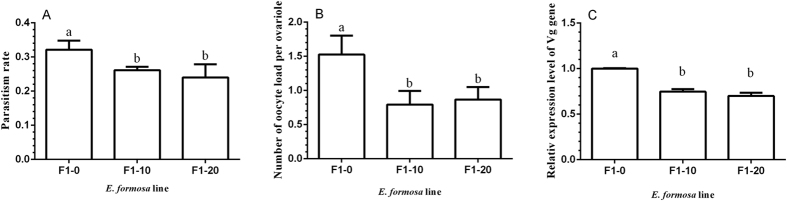
Reproductive effects of the *Wolbachia* titer on F1 lines. (**A**) Parasitism rate. (**B**) Oocyte load per ovariole. (**C**) Relative expression levels of vitellogenin gene. F1-0, F1-10, and F1-20 are the offspring of the females in the F0-0, F0-10, and F0-20 lines, respectively. The values are means + SD; means with different letters are significantly different (LSD test, *P *<* *0.05).

The parasitism rates of whitefly nymphs by F1-10 and F1-20 wasps were 26.13 ± 0.99% and 24.00 ± 3.89%, respectively, but were both lower than the parasitism rate of the control line (32.13 ± 2.64%) (Fig. 7A, F_2,12_ = 10.81, *P *<* *0.05). The oocyte load of F1-0 (1.5 ± 0.3) were more than that of F1-10 (0.8 ± 0.2) and F1-20 (0.9 ± 0.2) lines (Fig. 7B, F_2,42_ = 48.23, *P *<* *0.0001). The relative expression levels of the vitellogenin gene of the F1-10 and F1-20 lines were not significantly different, but both were lower than that of their control line (Fig. 7C, F_2,6_ = 109.85, *P *<* *0.0001). Because the 50 mg/ml treatment was too detrimental to the wasps; they were unable to survive past three days and produced few offspring. Therefore, the F1-50 wasps were not available to be tested.

### Female offspring proportions and *Wolbachia* infection rate

**Figure 8:**
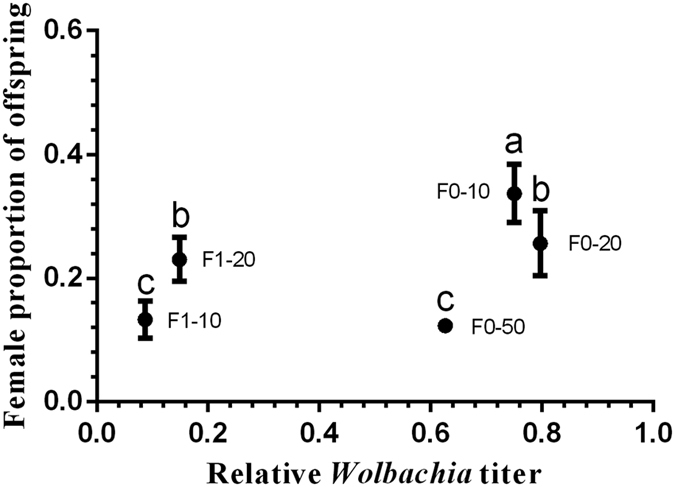
The relationship between the relative *Wolbachia* titers of wasps and the sex ratios of their offspring. F0-10, F0-20, and F0-50 are lines treated continuously with 10, 20, and 50 mg/ml tetracycline, respectively. F1-10 and F1-20 are the female offspring of the F0-10 and F0-20 lines, respectively. The values are means ± SD; means with different letters are significantly different (LSD test, *P* < 0.05).

After being fed tetracycline, females produced not only female but also male offspring. The female offspring proportion of F0 lines decreased significantly with the increase of tetracycline concentration, however, it was significantly higher for F1-20 than that for F1-10 (Fig. 8, F_4,20_ = 27.91, *P* < 0.0001). With the order of *Wolbachia* titer from high to low, female offspring proportions were 33.68 ± 4.71% (F0-10), 25.63 ± 5.27% (F0-20), 12.31 ± 0.56% (F0-50) for the F0 generation and 13.31 ± 2.96% (F1-10), and 23.03 ± 3.52% (F1-20) for the F1 generation.

All the female wasps were infected with *Wolbachia*, and in contrast to our prediction, some male offspring were infected with *Wolbachia*. The *Wolbachia* infection rates of male offspring were 43.5% (F0-10), 89.37% (F0-20), 74.90% (F0-50), 40.59% (F1-10), and 38.13% (F1-20).

## Discussion

To remove *Wolbachia*, the application of tetracycline in diet has been the most common method in those studies that have focused on the effects of *Wolbachia* on *E. formosa* reproduction^12^,^13^. However, when the reproductive phenomena induced by *Wolbachia* were evaluated, the effects of tetracycline were also involved. Here, we treated the F0 lines with different concentrations of tetracycline solution to obtain F1 lines with different *Wolbachia* titers. Excluding the effects of tetracycline, our results from the F1 lines strongly suggest that *Wolbachia* has positive effects on female fecundity. For instance, F1-0 wasps with high *Wolbachia* titers produced more progeny than the F1-10 and F1-20 wasps with low *Wolbachia* titers. Removing *Wolbachia* inhibited oogenesis in *Asobara tabida*^18^, and consistently here we found decreased oocyte loads in the F1-10 and F1-20 wasps in which *Wolbachia* titers were lower compared with those in *Wolbachia*-infected control line. In addition, oogenesis was positively ralated to vitellogenesis, and vitellogenin was shown to play an important role in wasp reproduction^19^,^23^. To explain the effects of *Wolbachia* on the molecular mechanism of wasp reproduction, we analyzed the relationship between *Wolbachia* and the expression of the vitellogenin gene and found that the expression was down-regulated after approximately 75% of *Wolbachia* were removed in the F1-10 and F1-20 lines. Thus, *Wolbachia* did enhance the fecundity of *E. formosa* probably through inducing upregulation of the vitellogenin gene.

In the present study, we used three different doses of tetracycline to treat the F0 wasps. The quantitative data showed that the *Wolbachia* titers were decreased after tetracycline treatment. Then we tested parasitism rate, oocyte load and vitellogenin gene expression to clarify variation in fertility and found that the fertility of the F0 lines—especially the F0-20 line—increased after treatment with tetracycline for 24 h. Because tetracycline reduced the *Wolbachia* titer and low titer *Wolbachia* is harmful to the wasps, the enhanced fertility could be caused by tetracycline. The increased fertility in cured host was also found by Zchori-Fein *et al*.^12^ and Stouthamer^13^. It is possible that treatment with tetracycline at a certain dose may have temporarily induced a stress response that stimulated wasp fertility. A similar result occurred when *E. formosa* were fed 1 mg/ml tetracycline for 24 h^14^. Moreover, we observed that long-term treatment with tetracycline has a detrimental effect on the wasps. The oocyte load of the treated wasps decreased in the days following the tetracycline treatments, and wasp longevity was also negatively affected. Stouthamer and Mak also found that high doses of tetracycline reduced wasp progeny production significantly over a long post-treatment period^13^. Consequently, these differing host responses to short-term and long-term treatments of antibiotic should be carefully considered in future evaluations of symbiont effects on hosts when antibiotics are used.

If there was a wasp population uninfected by *Wolbachia*, it would be easier to determine the effects of either tetracycline or *Wolbachia*. However, we failed to establish an uninfected population; at present, it is impossible to obtain stable *Wolbachia*-uninfected lines. Although the female wasps in the G3 generation were treated with a 10 mg/ml dose of tetracycline for 24 h, the reduction in female offspring and *Wolbachia* caused the population growth to fail, most of all, the *Wolbachia* still could be detected in *E. formosa* of fifth generation. Furthermore, the female wasps produced males only after several days post-treatment, and the resulting male wasps failed to inseminate female *E. formosa*^12^,^13^. It has been suggested that *Wolbachia* infection in this species occurred long time ago and the males had lost the ability to fertilize the females^12^. In neither previous studies^15^ nor in the present study did we observe any mating behaviour between the male and female wasps. Mostly, previous studies determined that *Wolbachia* were removed either by observing female production and determining whether *E. formosa* produced only male offspring or by conducting common PCR after tetracycline treatment^12^,^14^,^15^. However, our common PCR and quantitative data showed that all the female wasps—including the female offspring of F0-50 wasps which were treated with 50 mg/ml tetracycline solution—were still infected with *Wolbachia*. A dosage of 50 mg/ml tetracycline solution caused high wasp mortality in the present study (82.23%) and similar results in other studies involving *E. formosa*^13^,^14^,^15^. Furthermore, even some males were infected with *Wolbachia* (although the *Wolbachia* titers were much lower than those of females). Consequently, tetracycline can only partially remove *Wolbachia* from female wasps; they still produced *Wolbachia*-infected offspring. The failure in removing *Wolbachia* may result from the resistance of the strain of *Wolbachia* to tetracycline, which prevents protein synthesis^32^, however rifampicin that interferes with nucleic acid synthesis may work^33^,^34^.

We also found *Wolbachia* affected sex determination in *E. formosa*. The sex ratio of offspring shifted and became more male-biased when *Wolbachia* titers were decreased by treatment with antibiotic. The main sex determination in Hymenoptera is haplodiploidy, in which unfertilized eggs develop into haploid males and fertilized eggs develop into females^35^,^36^,^37^. Another common sex determination model proposed to explain complex sex determination systems in Hymenoptera is complementary sex determination (CSD), in which heterozygous diploid eggs develop into female and homozygous diploid or hemizygous halploid eggs develop into males, however, the diploid males are inviable or sterile^35^. The sex determination in thelytokous reproduction associated with bacteria is more complicated and the mechanism is not fully understood at present^16^,^36^,^38^. Haplodiploidy reproduction was restored in some insects with bacteria induced thelytoky after being treated with antibiotic^39^. In studies involving *E. formosa*^12^,^13^, including the present one, no effective mating has been found. Kajita^40^ reported the mating behavior of male with antibiotic treated and untreated female *E. formosa*, however, no information about the offspring were reported in the paper. This is different from that in *Trichogramma*^41^, in which arrhenotokous males could mate with thelytokous females and fertilize the eggs. In experiments with another species of the genus *Encarsia*, after being cured by tetracycline the *Cardinium*-induced thelytokous *E. hispida* produced diploid males, and sexual line could not be established^38^. So we assume that the males of *E. formosa* in the present study are homozygous diploid, and *Wolbachia* feminize the diploid males.

The FISH showed that fewer *Wolbachia* remained in the ovaries of the F1-10 females than in the ovaries of F0-10 females, and the female proportion of offspring of the former was lower than that of the latter. Therefore, the lower the *Wolbachia* titer is, the higher the proportion of male offspring is. We suggest that *Wolbachia* titers in the wasps are commonly higher than the threshold that produces rare males and that *Wolbachia* is abundant in females in nature. These levels benefit the vertical transmission of *Wolbachia* by inducing thelytokous reproduction mode in its host. In this manner, *Wolbachia* promotes the co-evolution of mutualism and impacts the population developments of its hosts. Furthermore, although the male offspring proportion increased with decrease of *Wolbachia* titer in parent, different from other hymenopteran insects, such as *Asobara japonica*^15^, the mating function of uninfected-male was lost in the present and previous studies^11^, indicating that the bacteria have evolved close relationship with *E. formosa*. We also found that *Wolbachia* present in *E. formosa* collected from four far apart locations in China are the same strain (*w*For) (our unpublished data) indicating a stable relationship between *Wolbachia* and *E. formosa*.

In conclusion, we used quantitative PCR to detect *Wolbachia* in this study, a method that is more accurate than the methods used in previous studies and discovered that tetracycline treatments are able to only partially remove *Wolbachia* from *E. formosa*. This study is also the first to demonstrate that male *E. formosa* are also infected by *Wolbachia*. In addition, although feeding on 10% tetracycline solution caused increased wasp fertility in 24 h, more than 24 h continuous feeding had negative effects on the fertility and longevity of the wasp. Our results clearly showed that *Wolbachia* are advantageous to wasp fecundity and that *Wolbachia* titers affect the sexual developments of wasp eggs.

## Methods

All experimental protocols were approved by Nanjing Agricultural University.

All methods were carried out in accordance with relevant guidelines and regulations.

### Insect and parasitoid cultures

The Q biotype *Bemisia tabaci* (MED species), hosts of *E. formosa*, were originally collected from tomato, *Lycopersicon esculentum*, on the campus of Nanjing Agricultural University. *Bemisia tabaci* biotype identification and polymerase chain reaction (PCR) detection of *Wolbachia* were based on Ji *et al*.^42^. *Wolbachia*-uninfected whiteflies (Mediterranean species) kept in growth chambers at 25 ± 1 °C, 70 ± 5% RH, and a16/8 L/D photoperiod were used as hosts for the *E. formosa* used in the following tests. The *Wolbachia*-infection status of whitefly was screened once every two or three generations.

The *E. formosa* culture was initially acquired from Beijing Ecoman Biotechnology Co., Ltd, as pupae inside the pupal remains of greenhouse whiteflies (*Trialeurodes vaporariorum*). Using sequencing 16S ribosomal DNA of bacteria^43^ and multilocus sequence typing^44^, *E. formosa* infected only with the *w*For *Wolbachia* strain were selected and maintained on *B. tabaci* on tomato plants for several generations.

### Select *Wolbachia*-uninfected *E. formosa*

Ten newly emerged female wasps were starved for 24 h and then fed with 1 ml 10% (w/w) sucrose solution mixed with 10 mg tetracycline hydrochloride (Sigma-Adlrich, St. Louis, USA) for 24 h. To produce the next generation, individual wasps were provided with more than 20 3^rd^-instar whitefly nymphs for 24 h. Then DNA was extracted from the female wasps to detect *Wolbachia* by PCR. Following the detection method of Zhou *et al*.^45^, the primers used were *wsp*-81F: 5′-TGG TCC AAT AAG TGA TGA AGA AAC-3′ and *wsp*-691R: 5′-AAA AAT TAA ACG CTA CTC CA-3′, producing a 599 bp amplicon. The experimental procedure was repeated for four generations (G0, G1, G2, and G3). Wasps of every generation were counted and sexed.

### Antibiotic treatments

Fifty milligrams of tetracycline hydrochloride (Sigma-Adlrich, St. Louis, USA) was mixed with a 1 ml 10% (w/w) sucrose solution to create a 50 mg/ml tetracycline solution. This solution was then diluted to obtain 10 and 20 mg/ml tetracycline solution. Pure 10% sucrose solution was used as control. Female wasps (F0), within 12 h of emergence were starved for 24 h. Then, the wasps were fed with pure 10% sucrose solution (control) or one of the three concentrations of tetracycline sucrose solution for several days until all the wasps died. After 24 h of tetracycline treatment, nine wasps from each treatment were selected and DNA was extracted for *Wolbachia* quantification. The parental wasp lines that were treated with pure sucrose solution or with the 10, 20, or 50 mg/ml concentrations of tetracycline solution were denoted as F0-0, F0-10, F0-20, and F0-50, respectively. To acquire filial wasps, each F0-0, F0-10 or F0-50 female was placed with dozens of 3^rd^-instar whitefly nymphs for 24 h. Based on their mothers from the F0 lines, the female filial wasps were denoted as F1-0, F1-10, F1-20, and F1-50, while male offspring were denoted as F1-10M, F1-20M, and F1-50M, respectively. Nine female wasps and all male wasps that were less than 24-h-old in the F1 lines were collect to extract DNA for *Wolbachia* quantification. To determine the effects of tetracycline on wasp longevity, 45 newly emerged F0 wasps from each of the four treatments were placed into cylindrical glass tubes (9 cm × 3 cm) and starved for 24 h. Then, they were continuously fed with the tetracycline treatment solutions. The number of dead wasps was recorded once every 24 h until all the wasps died. These experiments were carried out in growth chambers at 25 ± 1 °C, 70 ± 5% RH, and a 24 h dark cycle to prevent tetracycline from breakdown by light.

### Quantifications of *Wolbachia*

DNA was extracted from whole wasps using the Wizard^®^ SV Genomic DNA Purification System (Promega A2361; Promega Biotech, Beijing, Co., Ltd.) according to the manufacturer’s instructions for tissue samples. Quantitative PCR was used to quantify *Wolbachia* in individual wasps infected with *Wolbachia*, which was determined by performing PCR amplifying the *wsp* gene following Baldo *et al*.^44^. The primers used for quantification of *Wolbachia* are listed in Table S1. DNA was quantified with SYBR^®^ Premix Ex Taq^TM^ (Tli RNaseH Plus) (Takara Biotechnology, Dalian, Co., Ltd.) according to the manufacturer’s protocol. Samples were run in triplicate, and the relative quantities of *Wolbachia* were calculated based on the comparative cycle threshold 2^−ΔΔCt^ method^46^.

### FISH detection and location of *Wolbachia* in *E. formosa*

The FISH procedure to detect *Wolbachia* in female and male *E. formosa* followed the method of Zhao *et al*.^47^. Targeting the 16S rRNA of *Wolbachia*, two 5′ rhodamine-labelled *Wolbachia* probes, 5′-AAT CCG GCC GAR CCG ACC C-3′ (W1) and 5′-CTT CTG TGA GTA CCG TCA TTA TC-3′ (W2), described by Heddi *et al*.^48^ were used. Female wasps in the F0-0 line and both female and male wasps in the F1-10 line were randomly sampled. The wasp samples were stained and mounted, and *Wolbachia* infection were viewed under a ZEISS LSM 700 confocal microscope (Carl Zeiss, Germany).

### Parasitism rates and sex ratios

Twenty newly emerged whiteflies, uninfected by *Wolbachia*, were introduced into a 9-cm-diameter Petri dish containing a fresh tomato leaf in a growth chamber (25 ± 1 °C, 70 ± 5% RH, and 16/8 L/D). The end of the leaf petiole was covered with watered cotton to keep the leaf fresh. After 24 h, the adults were removed, and their eggs were monitored daily. Approximately ten days later, a pin was used to remove surplus nymphs until only fifty 3^rd^-instar nymphs remained on the leaf. Then, three *E. formosa* female wasps from the F0 or F1 lines were introduced into the Petri dish and removed 72 h later. The nymphs were checked once every 24 h, and emerged wasps were counted and sexed. The parasitism rate was calculated by dividing the number of emerged wasps by fifth, and female proportions were calculated by dividing the number of females by the total number of emerged wasps per Petri dish. Each line (F0 and F1) was replicated five times.

### Oocyte load

To count the number of oocytes, fifteen wasp specimens from each of the F0 or F1 lines were dissected under a stereomicroscope (Zeiss Discovery V12) in a phosphate buffer saline (PBS) (0.01 M, pH 7.3 ± 1). The parasitoids are ready for parasitization in 12 h after emergence, so wasps were sampled 24 h after emergence. The number of oocytes and the number of ovarioles in each female reproductive system were counted. The number of oocytes per ovariole was calculated by dividing the total oocytes by the number of ovarioles in each individual.

### Expression of vitellogenin gene

RNA extractions were performed on the F0 and F1 females. Total RNA was isolated from 100 whole-body females for each of the three replicates with a combination of TRIzol^®^ Reagent (Life Technologies, Carlsbad, CA) extraction. All RNA samples were further purified according to TRIzol^®^ manufacturer instructions. The cDNAs were synthesized by PrimeScript^TM^ RT Reagent Kit with gDNA Eraser (Takara Biotechnology), in accordance with the manufacturer’s instructions. The expression of vitellogenin gene in all samples was measured by q-PCR in triplicate, and primers are listed in Table S2. The relative transcript levels are expressed as the mean ± SD for each time point using the 2^−ΔΔCt^ method^46^.

### Statistical analysis

SPSS (version 13.0; SPSS Inc., Chicago, IL, USA) was used to perform the statistical analyses. One-way analysis of variance (ANOVA) was used to analyse the effect of tetracycline concentration on the *Wolbachia* titers using general linear model (GLM). The reproductive effects of tetracycline for the parameters parasitism rate, oocyte load, wasp longevity, and the relative expression levels of vitellogenin gene, and the effect of *Wolbachia* titer on female proportions were analysed by one-way ANOVAs using GLM. Data were first checked for normality and transformed when necessary to meet the assumption of normal distribution. The parasitism rates and female proportions were arcsine-square root transformed before being subjected to ANOVAs, and untransformed data were presented. The least significant difference (LSD) test was used to separate all means other than the means of two sets of oocyte load data, which were separated by using independent samples t-test. All graphs were constructed using GraphPad Prism 6 software (GraphPad Software, San Diego, CA, USA).

## Additional Information

**How to cite this article**: Wang, X.-X. *et al*. Incomplete removal of *Wolbachia* with tetracycline has two-edged reproductive effects in the thelytokous wasp *Encarsia formosa* (Hymenoptera: Aphelinidae). *Sci. Rep.* **7**, 44014; doi: 10.1038/srep44014 (2017).

**Publisher's note:** Springer Nature remains neutral with regard to jurisdictional claims in published maps and institutional affiliations.

## Supplementary Material

Supplementary Information

## References

[b1] Zug R. & Hammerstein P. Still a host of hosts for *Wolbachia*: analysis of recent data suggests that 40% of terrestrial arthropod species are infected. PLoS one 7, e38544 (2012). 22685581 10.1371/journal.pone.0038544 PMC3369835

[b2] Min K. T. & Benzer S. *Wolbachia*, normally a symbiont of *Drosophila*, can be virulent, causing degeneration and early death. Proc. Natl. Acad. Sci. USA 94, 10792–10796 (1997). 9380712 10.1073/pnas.94.20.10792 PMC23488

[b3] Dobson S. L. . *Wolbachia* infections are distributed throughout insect somatic and germ line tissues. Insect Biochem. Mol. Biol. 29, 153–160 (1999). 10196738 10.1016/s0965-1748(98)00119-2

[b4] Werren J. H., Baldo L. & Clark M. E. *Wolbachia*: master manipulators of invertebrate biology. Nat. Rev. Microbiol. 6, 741–751 (2008). 18794912 10.1038/nrmicro1969

[b5] Zug R. & Hammerstein P. Bad guys turned nice? A critical assessment of *Wolbachia* mutualisms in arthropod hosts. Biol. Rev. 90, 89–111 (2015). 24618033 10.1111/brv.12098

[b6] Vavre F., Girin C. & Bouletreau M. Phylogenetic status of a fecundity-enhancing Wolbachia that does not induce thelytoky in *Trichogramma*. Insect Mol. Biol. 8, 67–72 (1999). 9927175 10.1046/j.1365-2583.1999.810067.x

[b7] Stouthamer R. & Luck R. Influence of microbe-associated parthenogenesis on the fecundity of *Trichogramma deion* and *T. pretiosum*. Entomol. Exp. Appl. 67, 183–192 (1993).

[b8] Silva I. M. M. S. . Biological control potential of *Wolbachia*-infected versus uninfected wasps: laboratory and greenhouse evaluation of *Trichogramma cordubensis* and *T. deion* strains. Biocontrol Sci. Tech. 10, 223–238 (2000).

[b9] Zchori-Fein E., Gottlieb Y. & Coll M. *Wolbachia* density and host fitness components in *Muscidifurax uniraptor* (Hymenoptera: Pteromalidae). J. Invertebr. Pathol. 75, 267–272 (2000). 10843833 10.1006/jipa.2000.4927

[b10] Gelman D. B., Gerling D., Blackburn M. B. & Hu J. S. Host-parasite interactions between whiteflies and their parasitoids. Arch. Insect Biochem. Physiol. 60, 209–222 (2005). 16304614 10.1002/arch.20101

[b11] Hoddle M. S., Van Driesche R. G. & Sanderson J. P. Biology and use of the whitefly parasitoid *Encarsia formosa*. Annu. Rev. Entomol. 43, 645–669 (1998). 15012401 10.1146/annurev.ento.43.1.645

[b12] Zchori-Fein E., Roush R. & Hunter M. Male production induced by antibiotic treatment in *Encarsia formosa* (Hymenoptera: Aphelinidae), an asexual species. Experientia 48, 102–105 (1992).

[b13] Stouthamer R. & Mak F. Influence of antibiotics on the offspring production of the *Wolbachia*-infected parthenogenetic parasitoid *Encarsia formosa*. J. Invertebr. Pathol. 80, 41–45 (2002). 12234541 10.1016/s0022-2011(02)00034-4

[b14] Zhou S., Li Y. & Zhang F. Influence of *Wolbachia* on reproduction and the fitness of the parasitoid wasp *Encarsia formosa*. Acta Phytophy. Sin. 36, 7–10 (2009).

[b15] Tong L., Qi L. D., Zhang F. & Li Y. X. Effects of antibiotic treatment on reproduction of *Encarsia formosa* (Hymenoptera: Aphelinidae) infected with *Wolbachia*. Acta Entomol. Sin. 55, 933–940 (2012).

[b16] Ma W. J. . Diploid males support a two-step mechanism of endosymbiont-induced thelytoky in a parasitoid wasp. BMC Evol. Biol. 15, 84 (2015). 25963738 10.1186/s12862-015-0370-9 PMC4456809

[b17] Stouthamer R., Breeuwer J. A. & Hurst G. D. *Wolbachia pipientis*: microbial manipulator of arthropod reproduction. Annu. Rev. Microbiol. 53, 71–102 (1999). 10547686 10.1146/annurev.micro.53.1.71

[b18] Dedeine F. . Removing symbiotic *Wolbachia* bacteria specifically inhibits oogenesis in a parasitic wasp. Proc. Natl. Acad. Sci. USA 98, 6247–6252 (2001). 11353833 10.1073/pnas.101304298 PMC33453

[b19] Raikhel A. S. & Dhadialla T. Accumulation of yolk proteins in insect oocytes. Annu. Rev. Entomol. 37, 217–251 (1992). 1311540 10.1146/annurev.en.37.010192.001245

[b20] Donnell D. M. Vitellogenin of the parasitoid wasp, *Encarsia formosa* (Hymenoptera: Aphelinidae): gene organization and differential use by members of the genus. Insect Biochem. Mol. Biol. 34, 951–961 (2004). 15350614 10.1016/j.ibmb.2004.06.011

[b21] Renucci M. & Strambi C. Juvenile hormone levels, vitellogenin and ovarian development in *Acheta domesticus*. Experientia 39, 618–620 (1983).

[b22] Bownes M. Expression of the genes coding for vitellogenin (yolk protein). Annu. Rev. Entomol. 31, 507–531 (1986).

[b23] Tufail M. & Takeda M. Insect vitellogenin/lipophorin receptors: molecular structures, role in oogenesis, and regulatory mechanisms. J. Insect Physiol. 55, 88–104 (2009). 10.1016/j.jinsphys.2008.11.007 19071131

[b24] Dong S. Z. . Vitellin of *Pteromalus puparum* (Hymenoptera: Pteromalidae), a pupal endoparasitoid of *Pieris rapae* (Lepidoptera: Pieridae): Biochemical characterization, temporal patterns of production and degradation. J. Insect Physiol. 53, 468–477 (2007). 17368664 10.1016/j.jinsphys.2007.01.008

[b25] Kawakami Y., Goto S. G., Ito K. & Numata H. Suppression of ovarian development and vitellogenin gene expression in the adult diapauses of the two-spotted spider mite *Tetranychus urticae*. J. Insect Physiol. 55, 70–77 (2009). 19022260 10.1016/j.jinsphys.2008.10.007

[b26] Guo J. Y. . Enhanced vitellogenesis in a whitefly via feeding on a begomovirus-infected plant. PloS one 7, e43567 (2012). 22937062 10.1371/journal.pone.0043567 PMC3427354

[b27] Attardo G. M., Hansen I. A., Shiao S. H. & Raikhel A. S. Identification of two cationic amino acid transporters required for nutritional signaling during mosquito reproduction. J. Exp. Bot. 209, 3071–3078 (2006). 10.1242/jeb.02349 16888056

[b28] Dong S. Z. . Effects of starvation on the vitellogenesis, ovarian development and fecundity in the ectoparasitoid, *Nasonia vitripennis* (Hymenoptera: Pteromalidae). Insect Sci. 15, 429–440 (2008).

[b29] Dong S. Z., Ye G. Y., Guo J. Y. & Hu C. Roles of ecdysteroid and juvenile hormone in vitellogenesis in an endoparasitic wasp, *Pteromalus puparum* (Hymenoptera: Pteromalidae). Gen. Comp. Endocr. 160, 102–108 (2009). 19032957 10.1016/j.ygcen.2008.11.007

[b30] Ye G. Y. . Effects of host (*Boettcherisca peregrina*) copper exposure on development, reproduction and vitellogenesis of the ectoparasitic wasp, *Nasonia vitripennis*. Insect Sci. 16, 43–50 (2009).

[b31] Caragata E. P., Rancès E., O’Neill S. L. & McGraw E. A. Competition for amino acids between *Wolbachia* and the mosquito host, *Aedes aegypti*. Microb. Ecol. 67, 205–218 (2014). 24337107 10.1007/s00248-013-0339-4

[b32] Li Y. Y., Floate K. D., Fields P. G. & Pang B. P. Review of treatment methods to remove *Wolbachia* bacteria from arthropods. Symbiosis. 62, 1–55 (2014).

[b33] Pike N. & Kingcombe R. Antibiotic treatment leads to the elimination of *Wolbachia* endosymbionts and sterility in the diplodiploid collembolan *Folsomia candida*. BMC Biol. 7, 54 (2009). 19698188 10.1186/1741-7007-7-54 PMC2739161

[b34] Li Y. Y., Fields P. G., Pang B. P. & Floate K. D. Effects of tetracycline and rifampicin treatmens on the fecundity of the *Wolbachia*-infected host, *Tribolium confusum* (Coleoptera: Tenebrionidae). J. Econ. Entomol. 109, 1458–1464 (2016). 10.1093/jee/tow067 27114607

[b35] Heimpel G. E. & de Boer J. G. Sex determination in the Hymenoptera. Annu. Rev. Entomol. 53, 209–230 (2008). 17803453 10.1146/annurev.ento.53.103106.093441

[b36] Verhulst E. C., Beukeboom L. W. & van de Zande L. Maternal control of haplodiploid sex determination in the wasp. Nasonia. Science 328, 620–623 (2010). 20431014 10.1126/science.1185805

[b37] Gottlieb Y. Check out these males. Heredity 103, 1–2 (2009). 19352405 10.1038/hdy.2009.35

[b38] Giorgini M., Monti M. M., Caprio E., Stouthamer R. & Hunter M. S. Feminization and the collapse of haplodiploidy in an asexual parasitoid wasp harboring the bacterial symbiont Cardinium. Heredity 102, 365–371 (2009). 19190669 10.1038/hdy.2008.135

[b39] Stouthamer R., Luck R. F. & Hamilton W. D. Antibiotics cause parthenogenetic *Trichogramma* (Hymenoptera/Trichogrammatidae) to revert to sex. Proc. Natl. Acad. Sci. USA 87, 2424–2427 (1990). 11607070 10.1073/pnas.87.7.2424 PMC53701

[b40] Kajita H. Induction of males in the thelytokous wasp *Encarsia formosa* Gahan (Hymenoptera: Aphelinidae). Appl. Entomol. Zoo. 28, 115–117 (1993).

[b41] Stouthamer R. & Kazmer D. J. Cytogenetics of microbe-associated parthenogenesis and its consequences for gene flow in *Trichogramma* wasps. Heredity 73, 317–327 (1994).

[b42] Ji H. L., Qi L. D., Hong X. Y., Xie H. F. & Li Y. X. Effects of Host Sex, Plant Species, and Putative Host Species on the Prevalence of *Wolbachia* in Natural Populations of *Bemisia tabaci* (Hemiptera: Aleyrodidae): A Modified Nested PCR Study. J. Econ. Entomol. 108, 210–218 (2015). 26470122 10.1093/jee/tou004

[b43] Weisburg W. G., Barns S. M., Pelletier D. A. & Lane D. J. 16S ribosomal DNA amplification for phylogenetic study. J. Bacteriol. 173, 697–703 (1991). 1987160 10.1128/jb.173.2.697-703.1991 PMC207061

[b44] Baldo L. . Multilocus sequence typing system for the endosymbiont *Wolbachia pipientis*. Appl. Environ. Microb. 72, 7098–7110 (2006). 10.1128/AEM.00731-06 PMC1636189 16936055

[b45] Zhou W., Rousset F. & O’Neill S. Phylogeny and PCR-based classification of *Wolbachia* strains using wsp gene sequences. Proc. R. Soc. London, Ser. B 265, 509–515 (1998). 10.1098/rspb.1998.0324 PMC1688917 9569669

[b46] Livak K. J. & Schmittgen T. D. Analysis of relative gene expression data using real-time quantitative PCR and the 2^−ΔΔCT^ method. Methods 25, 402–408 (2001). 11846609 10.1006/meth.2001.1262

[b47] Zhao D. X., Zhang X. F., Chen D. S., Zhang Y. K. & Hong X. Y. *Wolbachia*-host interactions: host mating patterns affect *Wolbachia* density dynamics. PloS ONE 8, e66373, doi: 10.1371/jo-urnal.pone.0066373 (2013). 23823081 PMC3688896

[b48] Heddi A., Grenier A. M., Khatchadourian C., Charles H. & Nardon P. Four intracellular genomes direct weevil biology: nuclear, mitochondrial, principal endosymbiont, and *Wolbachia*. Proc. Natl. Acad. Sci. USA 96, 6814–6819 (1999). 10359795 10.1073/pnas.96.12.6814 PMC21998

