# Supplementary material for: Incomplete removal of Wolbachia with tetracycline has two-edged reproductive effects in the thelytokous wasp Encarsia formosa (Hymenoptera: Aphelinidae)
Source: Sci Rep. 2017 Mar 7;7:44014. doi: 10.1038/srep44014 (PMC5339822; doi:10.1038/srep44014)
Supplement: Supplementary Information [file srep44014-s1.pdf]

# Incomplete removal of *Wolbachia* with tetracycline has two-edged reproductive effects in the thelytokous wasp *Encarsia formosa* (Hymenoptera: Aphelinidae)

Xiao-Xiang Wang[1], Lan-Da Qi[1], Rui Jiang[1], Yu-Zhou Du [2], Yuan-Xi Li [1*]

1. Department of Entomology, Nanjing Agricultural University, Nanjing, Jiangsu 210095, China.

2. Institute of Applied Entomology, School of Horticulture and Plant Protection, Yangzhou University, Yangzhou, Jiangsu 225009, China.

Corresponding author: yxli@njau.edu.cn

**Figure S1**

***Wolbachia* detection of the individual females in five generations by PCR using *wsp* gene. A: the parental generation (G0, n = 10). B: G1 generation (n = 13). C: G2 generation (n = 6). D: G3 generation (n=7). E: G4 generation (n = 3; 1-3, 4-6 and 7-9 were three female samples technically repeated PCR testing in triplicate). M: DNA marker. *W*+: positive control. *W*-: negative control.**

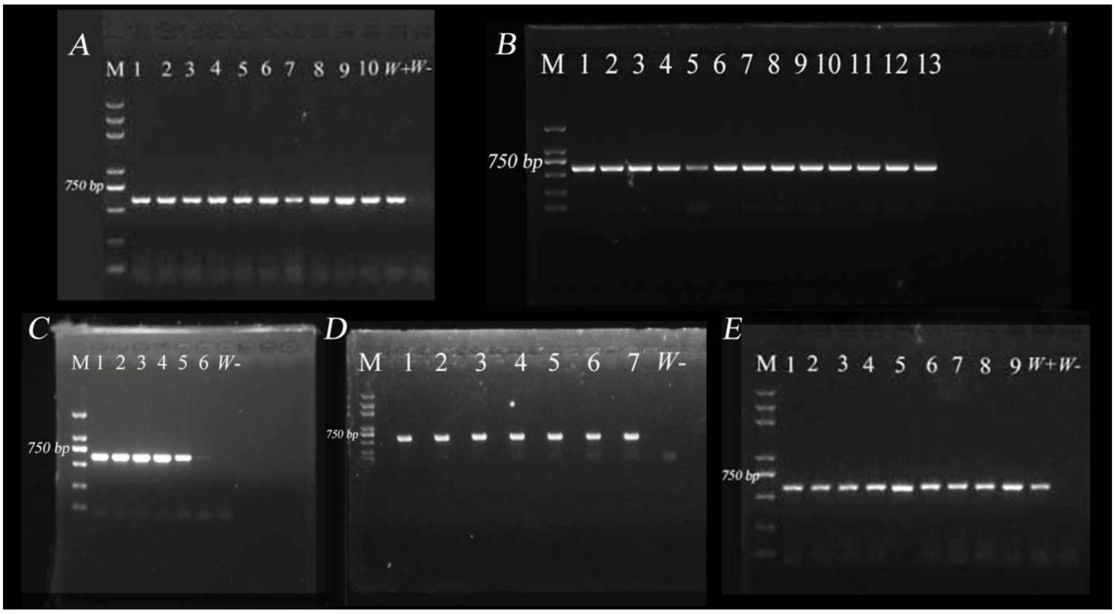

**Table S1** Primer sequences used for q-PCR analysis of *Wolbachia* quantification

| Gene | | Primer | Primer sequence (5' to 3') | Product Size (bp) |
|---|---|---|---|---|
| Target gene | *ftsZ* of *Wolbachia* | *ftsZ*-F | GGTGCTTTGCCTGATGTTG | 166 |
| | | *ftsZ*-R | CCGCTCTTGCTTCTCTGG | |
| Reference gene | 28S rRNA of *E. formosa* | 28S rRNA-F | CGCCACGAGACCGATCGC | 152 |
| | | 28S rRNA-R | GTAAGCCAAAGAGGTTGACGATG | |
| | Vitellogenin of *E. formosa* | Vg-F | CCCGCTCCTTCCGTTCTAC | 143 |
| | | Vg-R | TCCGCTCTTCCACCATACTATC | |

**Table S2** Primer sequences used for q-PCR analysis of vitellogenin gene expression

| Gene | | Primer | Primer sequence (5’ to 3’) | Product Size (bp) |
|---|---|---|---|---|
| Target gene | Vitellogenin | mVg-F | ACAATCCTTACTCGGCTCCTCAGA | 149 |
| | | mVg-R | TGCCTGAGCTTCGAGGAGATGA | |
| Reference gene | β-tubulin | β-tubulin-F | CATTGTGAGTGAAACGAGATGGTTGAG | 117 |
| | | β-tubulin-R | AACACCGACGAGACCTATTGTATTGAC | |
